# Accelerated Bacterial Identification with MALDI-TOF MS Leads to Fewer Diagnostic Tests and Cost Savings

**DOI:** 10.3390/antibiotics13121163

**Published:** 2024-12-02

**Authors:** Miriam Uzuriaga, Francisco Guillén-Grima, Marta Rua, José Leiva, José R. Yuste

**Affiliations:** 1 Clinical Microbiology Service, Clínica Universidad de Navarra, 31008 Pamplona, Spain; miriamuzuriaga@hotmail.com (M.U.); mrua@unav.es (M.R.); jleiva@unav.es (J.L.); 2 Prehospital Emergency Medical Service of Madrid Community, SUMMA112, 28045 Madrid, Spain; 3 Healthcare Research Institute of Navarre (IdiSNA), 31008 Pamplona, Spain; jryuste@unav.es; 4 Department of Preventive Medicine, Clínica Universidad de Navarra, 31008 Pamplona, Spain; 5 CIBER in Epidemiology and Public Health (CIBERESP), Institute of Health Carlos III, 46980 Madrid, Spain; 6 Department of Health Sciences, Public University of Navarra, 31008 Pamplona, Spain; 7 Service of Infectious Diseases, Clínica Universidad de Navarra, 31008 Pamplona, Spain; 8 Department of Internal Medicine, Clínica Universidad de Navarra, 31008 Pamplona, Spain

**Keywords:** clinical impact, economic impact, fast information, diagnostics, MALDI-TOF MS, costs

## Abstract

Introduction: Rapid microbiology reporting can enhance both clinical and economic outcomes. Material and Methods: This three-year, quasi-experimental study, single-group pretest–posttest study, conducted at a university medical center, aimed to evaluate the clinical and economic impact of rapid microbiological identification reporting using MALDI-TOF MS. A total of 363 consecutive hospitalized patients with bacterial infections were evaluated, comparing a historical control group (CG, n = 183) with an intervention group (IG, *n* = 180). In the CG, microbiological information (bacterial identification and antibiotic susceptibility) was provided between 18:00 and 22:00 h, while in the IG, bacterial identification was reported between 12:00 and 14:00 h, and antibiotic susceptibility was reported between 18:00 and 22:00 h. Results: The IG demonstrated a significant reduction in the number of patients undergoing Microbiology (*p* = 0.01), Biochemistry (*p* = 0.05), C-Reactive Protein (*p* = 0.02), Radiological Tests (*p* = 0.05), Computed Tomography Tests (*p* = 0.04), and Pathology (*p* = 0.01). However, no statistically significant reduction was observed in economic costs related to microbiological testing (*p* = 0.76) or antibiotic consumption (*p* = 0.59). The timely reporting of microbiological identification to clinicians resulted in fewer patients undergoing additional diagnostic tests, ultimately contributing to reduced healthcare resource utilization without adversely affecting clinical outcomes.

## 1. Introduction

Previous research [1] laid the foundation for demonstrating the clinical benefits of accelerated bacterial identification using MALDI-TOF MS. That study observed significant reductions in reporting times and early optimization of antibiotic prescribing without compromising clinical outcomes. The current research aims to build upon those findings through a comprehensive economic analysis, evaluating the cost-effectiveness of implementing MALDI-TOF MS.

To further connect this study with antibiotics use and antimicrobial stewardship, it is essential to note that implementing MALDI-TOF MS not only accelerated bacterial identification but also provided earlier data that allowed for more precise and timely optimization of antibiotic treatment [2,3,4]. This early intervention addresses the critical need for enhanced antimicrobial stewardship by reducing unnecessary or prolonged antibiotic courses—key contributors to antimicrobial resistance [2,4,5]. Faster reporting times, from sample collection to antibiotic susceptibility data, ensured that effective and targeted antibiotics could be administered sooner, thus promoting more rational antibiotic use [1,3].

Early and accurate microbiological diagnosis improves antibiotic prescribing and reduces inappropriate antibiotic use, which is crucial for controlling bacterial resistance [2,4]. However, achieving this goal requires rapid, reliable, and cost-effective diagnostics, which increases laboratory costs [6]. Considering the practical and economic constraints, it is essential to strike a balance between accuracy, speed, and affordability in diagnostics [7,8].

We are currently facing severe problems of bacterial resistance due to inappropriate antibiotic use [9,10]. More than 50% of prescriptions are unnecessary, exacerbating resistance and increasing healthcare costs [11,12,13,14]. Furthermore, the spread of multidrug-resistant bacteria represents a significant global threat, with projections for 2050 estimating that resistant infections will cause more deaths than cancer [15,16,17,18,19,20]. Antimicrobial resistance is a crucial concern for healthcare systems, as it increases morbidity mortality, prolongs hospital stays, and raises overall healthcare costs [20,21,22,23,24].

Implementing the MALDI-TOF MS system has decreased microbiological identification times and improved infection management by reducing response times and costs while optimizing antimicrobial therapy [1,25,26,27]. However, without an antimicrobial optimization program, MALDI-TOF MS may not improve treatment accuracy in settings with high rates of antibiotic resistance [28]. This underscores the importance of complementing advanced technologies with comprehensive antimicrobial stewardship strategies [28]. MALDI-TOF MS can reduce clinical failure rates, adverse events, and hospitalization costs [3,27,29,30]. Recent advances in mass spectrometry have improved the identification of many previously difficult microorganisms. Its application has progressively expanded to various bacterial genera, fungi, and mycobacteria [31,32].

The healthcare system faces significant challenges, including the rise in nosocomial infections, antimicrobial resistance, and the high cost of healthcare [18,33,34]. In this context, microbiology laboratories are essential for the appropriate use of antibiotics, allowing proactive measures and adjustments to treatments based on accurate identification of resistance mechanisms, thereby reducing comorbidity and length of hospital stay [20]. Developing effective strategies to identify bacteria and administer antibiotics is crucial to improving their efficacy and controlling the spread of nosocomial infections caused by multidrug-resistant microorganisms [29,35,36]. Furthermore, antimicrobial resistance and inappropriate antibiotic use represent a considerable burden on hospital budgets. Microbiology laboratories thus play a key role in reducing clinical failures and costs, improving treatment accuracy, and minimizing adverse patient effects [18,20,33,34]. Therefore, this study aims to observe the impact of rapid information in clinical terms—specifically the number of patients undergoing complementary tests—and in economic terms.

## 2. Results

In total, 363 consecutively hospitalized patients with documented bacterial infections were enrolled, divided into a control group (CG, *n* = 183) and an intervention group (IG, *n* = 180). The division of groups was based on the timeline of microbiological identification practices, with the CG representing the period before the implementation of rapid reporting and the IG representing the post-implementation phase.

### 2.1. Demographics and Clinical Basal Characteristics of Patients

The patients in this study have been described previously [1], which provides a detailed characterization of the population analyzed. Both groups were comparable in terms of the McCabe–Josson criteria (severity of underlying illness) [37] and the Charlson score (comorbidity index) [38]. The only notable difference was a higher proportion of patients under surgical care in the IG [1].

The distribution of infectious syndromes across the study groups is summarized in Table 1. Urinary infections were the most frequent, accounting for 37.7% of cases, with no significant difference between the control group (40.4%) and the intervention group (35.0%) (*p* = 0.28). Respiratory infections were observed in 27.6% of patients, including a higher proportion of pneumonia cases in the control group (12.6%) compared to the intervention group (4.4%) (*p* < 0.01). Skin and soft tissue infections were significantly more prevalent in the intervention group (22.2%) compared to the control group (13.7%) (*p* = 0.03). Other syndromes, such as abdominal, osteoarticular, and endovascular infections, showed no significant differences between groups. These findings provide insight into the heterogeneity of infection types and their potential impact on diagnostic and treatment strategies.

### 2.2. Samples and Infectious Syndrome

The mean time from sample collection to notification of index-positive culture (IPC) to the clinician was significantly shorter in IG (8.3 h) compared to the CG (29.9 h vs. 38.2 h; *p* ≤ 0.01. The report was made by telephone and via the computerized laboratory information system. IPC is defined as the first sample that shows bacterial growth. Furthermore, the mean time from the detection of bacterial growth to reporting of identification results to the clinician was significantly shorter (7 h) in the IG compared to CG (4.5 h vs. 11.4 h; *p* < 0.01). However, no significant difference was found between sample processing and detection of bacterial growth between the groups (25 h in IG vs. 25.6 h in CG; *p* = 0.14) [1].

Regarding infectious syndromes, the number of patients with a clinical diagnosis of pneumonia was significantly higher in the CG (*p* < 0.01), while skin and soft tissue infections were more frequent in the IG (*p* = 0.03) [1].

The most frequently processed samples included urine, lower respiratory tract samples, blood cultures, wounds, and abscesses. No significant differences were observed between the groups in the type of IPC [1].

### 2.3. Complementary Tests

We assess whether the availability of this rapid microbiological information to clinicians impacts the number of patients undergoing tests (microbiological, hematological, biochemical, and radiological) performed on patients. In terms of the percentage of patients undergoing these tests from the IPC, a lower decrease in the percentage of attendance is observed in the IG than in the CG, being significant in microbiological tests, biochemical tests, C-reactive protein, hematological tests, X-ray, CT scan, CT scan of the abdomen and anatomopathological tests, as detailed in Table S1.

### 2.4. Economic Analysis

An economic cost analysis considered direct fixed, direct variable, indirect, and total costs. The calculation was based on the consumer price index (CPI) of 2017, the year of the last patients included in the study (2017). The consumption of the different hospital resources was considered.

No differences were observed in the mean overall cost of care per patient in both groups (EUR 12,488.0 ± 8410.6 in CG patients vs. EUR 13,852.7 ± 8405.1 in IG patients; *p* = 0.12). There were also no significant differences between the groups related to the mean cost of global hospital stay (EUR 5204.2 ± 6689.6 in the CG and EUR 6899.4 ± 13,520.4 in the IG; *p* = 0.13) and the mean cost of the complementary examinations requested (microbiology laboratory [EUR 245.1 ± 315.8 in the CG and EUR 236.0 ± 265.1 in the IG; *p* = 0. 76]. The mean cost of non-microbiology laboratory was EUR 210.1 ± 350.5 in the CG and EUR 260.8 ± 447.5 in the IG (*p* = 0.23). Moreover, there were also no significant differences in non-laboratory examinations such as radiography, ultrasound, CT, and MRI [EUR 630.3 ± 1030.8 in the CG and EUR 650.1 ± 1096.2 in the IG; *p* = 0.85]) (Table 2 and Table 3).

The intervention group tended to reduce costs associated with antibiotics, although the decrease was not statistically significant. This reduction aligns with improved antimicrobial stewardship, where accelerated identification enables earlier adjustment of antibiotic therapy, either by narrowing broad-spectrum treatments or discontinuing unnecessary antibiotics altogether. Specifically, the lower mean cost of antibiotics in the IG compared to the CG (EUR 633.0 ± 859.1 vs. 698.1 ± EUR 1394.8, *p* = 0.59) supports the idea that faster bacterial identification may influence more judicious antibiotic use. Although no significant cost difference was observed, the trend suggests improved resource optimization.

When evaluating the average cost in pharmacy per patient although there are no significant differences between the two groups, it is lower in the IG than in the CG (EUR 3508.4± 2334.8 in CG patients vs. EUR 3198.5 ± 2606.8 in IG patients; *p* = 0.23) as well as in the antibiotics group (EUR 698.1 ± 1394.8 in CG patients vs. EUR 633.0 ± 859.1 in IG patients; *p* = 0.59). In the category of ‘other anti-infectives’, which includes the costs of treatment with antiparasitics, antimycobacterial, vaccines, antiseptics, disinfectants, immune sera, and immunoglobulins, no significant differences were found (*p* = 0.24) (Table 1).

The ‘other costs’ category included costs associated with clinical care, blood bank, surgery, and anesthesia, and we also found no statistically significant differences (EUR 2480.6 ± 4563.3 in CG patients vs. EUR 2371.9 ± 3688.7 in IG patients; *p* = 0.80) (Table 2 and Table 3).

## 3. Discussion

This study complements our previous research [1], which focused primarily on the clinical impact of rapid bacterial identification using MALDI-TOF MS. By conducting a detailed economic analysis, we demonstrated that the benefits of MALDI-TOF MS implementation extend beyond clinical outcomes, also resulting in cost savings related to reduced diagnostic testing and antibiotic consumption. Unlike most previous studies, which focus on specific sample types such as blood [12,26,27,28,39,40], urine [3,12,39,41], cerebrospinal fluid, [28,40,42] vitreous humor [43], and ascitic fluid [28,42,44], our research evaluated the impact of early diagnosis across a broader spectrum of clinical samples [1].

In previous studies by our group [12,13], we compared information on bacterial identification and antimicrobial susceptibility provided by the Vitek^®^ 2 system (bioMerieux, Marcy-l’Étoile, France) between 18:00 and 22:00 to the same information provided the following day, observing a 17.4-h reduction in identification reporting time. Consequently, Galar et al. [12,13] observed a significant reduction in microbiological, biochemical, and C-reactive protein tests. In our current study, we compared identification data obtained using Vitek^®^ 2 (bioMerieux, Marcy-l’Étoile, France) in the control group (CG) with data from Vitek^®^ MS (bioMerieux, Marcy-l’Étoile, France) in the intervention group (IG), achieving a time reduction of 8.3 h [1]. The MALDI-TOF MS system offers faster identification compared to Vitek^®^ 2 (bioMerieux, Marcy-l’Étoile, France), which expedites result delivery. In addition, MALDI-TOF MS provides higher accuracy and faster microbial identification due to the continuous updates of bacterial protein spectra [1,28,40,45,46,47,48,49,50,51,52]. Similar to our previous study [1], both groups in this study had antimicrobial susceptibility data available from 18:00 to 22:00 h. By working with various samples, we were able to assess the realistic clinical and economic impact of rapid bacterial identification.

When evaluating the total number of patients who underwent microbiology laboratory tests from IPC, significantly fewer patients were found in the IG. We used the number of patients requiring additional tests as a direct measure of the clinical impact of the intervention. Specifically, fewer patients underwent microbiological testing in the IG (136 [74.3%] patients in CG and 93 [51.7%] in IG; *p* = 0.01) (Table S1). These results align with findings from Doern et al. [39], who reported significant differences in patients undergoing microbiological testing, whereas Galar et al. [12] found a non-significant reduction in the number of patients who required such tests. These findings conclude that early reporting of microbiological identification leads to fewer patients undergoing microbiological testing.

These results underscore the importance of advanced diagnostics like MALDI-TOF MS for rapid pathogen identification and as a pivotal tool for optimizing antibiotic therapy. By providing earlier microbiological data, clinicians in the intervention group could adjust antibiotic treatments more rapidly, potentially decreasing the duration of unnecessary empirical antibiotic use. The reduction in the number of complementary tests among the intervention group patients could also indicate a more accurate early diagnosis and adjustment of antibiotic therapy, which limited the progression of infections that would otherwise necessitate further diagnostic evaluations. This finding highlights the critical role of microbiology services in antibiotic stewardship efforts, facilitating the reduction of inappropriate antibiotic use and contributing to controlling antimicrobial resistance.

This study’s distribution of infectious syndromes highlights key differences between the control and intervention groups that may influence diagnostic and economic outcomes. For example, the higher prevalence of pneumonia in the control group and the greater proportion of skin and soft tissue infections in the intervention group reflect variability in clinical presentations (Table 1). These differences could account for variations in test utilization and treatment costs, as respiratory and skin infections often require distinct diagnostic approaches. Although these findings did not result in significant differences in overall costs, they underscore the importance of considering the type of infection when evaluating the clinical and economic impact of interventions like MALDI-TOF MS. Future studies should explore these dynamics further, particularly in multi-center settings, to better generalize findings across diverse patient populations.

Regarding the number of patients undergoing non-microbiology laboratory tests, we found differences in the IG (161 [88.0%] patients) vs. the CG (145 [80.6%] patients; *p* = 0.05), within which we also found significantly fewer patients in the IG in the number of patients undergoing biochemical tests (145 [80.6%] patients) vs. the CG (161 [88.0%] patients); *p* = 0.05 (Table S1). These differences were observed by Galar et al. [12] and Doern et al. [39], who also found significant differences highlighting the importance of providing microbiological identification information early, considering that in our work, the identification information is provided earlier, thus impacting on the number of patients undergoing biochemical testing.

Among patients undergoing biochemical testing, it is noteworthy that patients undergoing C-reactive protein determinations are significantly lower in the IG (148 [80.9%] patients in the CG and 127 [70.6%] patients in the IG; *p* = 0.02) (Table S1). These findings differ from those described by Galar et al. [12], who observed a non-significant decrease in C-reactive protein in the IG.

In addition, probably due to early microbiological identification like Galar et al. [12], we observed a lower total number of patients undergoing hematological laboratory tests in the IG compared to the CG (172 [94.0%] patients in the CG and 158 [85.6%] patients in the IG; *p* = 0.04) more specifically concerning the number of patients who undergo hemograms in the IG compared to the CG (157 [85.8%] patients in the CG and 141 [78.3%] patients in the IG; *p* = 0.06), although not statistically significantly (Table S1). Galar et al. [12] found no significant differences regarding hemograms compared to Doern et al. [39], who observed fewer patients with hemograms in the IG.

We observed, due to the advancement of microbiological identification information [12], statistically significant differences in the total number of patients undergoing non-laboratory examinations between the two groups (142 [77.6%] patients in the CG and 109 [60.6%] patients in the IG; *p* = 0.01) (Table S1). Within this section, when analyzing the total number of patients undergoing X-rays we found significant differences in the IG compared to CG (100 [54.6%] patients in the CG and 80 [44.4%] patients in the IG; *p* = 0.05), CT (44 [24.0%] patients in the CG and 28 [15.5%] patients in the IG; *p* = 0.04), abdominal CT (30 [16.4%] patients in the CG and 17 [9.4%] patients in the IG; *p* = 0.05) and pathology tests (10 [5.5%] patients in the CG and 1 [0.6%] patients in the IG; *p* = 0.01) (Table S1). These data coincide with the findings of Galar et al. [12] regarding pathology in the significant reduction, although not in the case of X-rays, CT, and abdominal CT, where, although they found a lower number in the IG, it was not significant. Doern et al. [38] also observed significant differences in the number of radiological tests, with fewer tests being conducted in their intervention group. They did not find significant differences in CT scans, although a lower number was observed in their IG, and they did not provide information on pathological anatomy. The lesser impact observed in their study may be attributed to a longer identification reporting time compared to ours. We also found no significant differences between the two groups’ mean number of plain ultrasound and MRI scans. Like Galar et al. [12], we found no significant differences in the mean number of nuclear, cardiovascular, respiratory, and digestive tests or the total number of such tests. Doern et al. [39] did not report on these aspects. The decrease in number of patients undergoing X-rays, CT scans, abdominal CT scans, and pathology tests in our study could be attributed to the prompt availability of microbiological results, which enabled clinicians to make earlier decisions, thus avoiding the need for additional diagnostic and complementary tests. In addition, the reduction in the number of patients requiring such tests could reflect a more favorable clinical progression in the IG.

Good healthcare management should optimize financial resources and provide quality care. Several studies (Bruins et al. [53], Doern et al. [39], Galar et al. [12], Tran et al. [54], Ge et al. [55]) analyzed the economic impact of rapid microbiological information. Our study adjusted costs to December 2017 using the Bank of Spain’s dollar–euro conversion and Consumer Price Index (CPI).

Bruins et al. [53] did not find a significant economic impact using Vitek^®^ 2 due to the distance between the laboratory and the hospital and the lack of a hospital IT system. In contrast, studies in the USA (Kerremans et al. [42], Doern et al. [39]) did report a significant impact attributed to the proximity of the laboratory to the hospital and the presence of a hospital IT system. Galar et al. [12], with similar resources to ours, also found significant differences.

Patel et al. [3] and Perez et al. [30] evaluated the MALDI-TOF MS system, finding cost savings in IG compared to CG. Our study’s mean total cost per patient was EUR 12,488.0 in CG and EUR 13,852.7 in IG (Table 1). Although the results of Perez et al. [30] were similar, the differences in ICU (18.1% in our study vs. 42.8% in Perez et al. [30]) could explain the discrepancies in costs. Patel et al. [3] reported mean ICU costs of EUR 12,329.6 in the CG and EUR 9730.2 in the IG.

Regarding hospital stay costs, there was no statistical significance in our study (EUR 5204.2 in the CG vs. EUR 6899.4 in the IG; *p* = 0.13) (Table 1). In contrast, Galar et al. [12] found a significant reduction in the IG (*p* ≤ 0.01). Differences in microbiological identification time (6.9 h in our study vs. 17.6 h in Galar et al. [12]) could explain the lower significance.

Ge et al. [55] and Tran et al. [54] observed cost reductions with Vitek^®^ MS compared to Vitek^®^ 2, which was also reflected in our study, although the difference was not statistically significant (EUR 245.1 in CG vs. EUR 236.0 in IG; *p* = 0.76).

The lower cost observed in our study, both in pharmacy and within this in the cost of antibiotics, could be related to early optimization of antibiotic therapy facilitated by early microbiological identification and better resource management. It is relevant to note that urinary tract infections prevailed in our population, similar to the study by Doern et al. [39]. In contrast, Galar et al. [12] and Patel et al. [3] found a predominance of catheter-related bacteraemias, surgical site infections, and intra-abdominal infections. This difference in the type of infections could explain the lower antibiotic cost in our study compared to that of Galar et al. [12] and Patel et al. [3]. In addition, the study by Patel et al. [3] was conducted in the United States, where drug prices are approximately 2.8 times higher than in other countries such as Spain [56,57]. It is also important to note that the Doern et al. study [39] was conducted in 1994, while ours was conducted in 2017 when awareness of resource rationalization was more prominent. Resource rationalization brings not only clinical benefits, such as a reduction in potential medication errors, but also economic benefits, such as a decrease in cost per patient [58].

Our study defined ‘other costs’ as those associated with clinical care, blood bank, surgery, and anesthesia. We observed a trend towards lower cost in IG compared to CG, although this difference was not statistically significant (EUR 2480.6 in CG and EUR 2371.9 in IG; *p* = 0.80) (Table 2). In contrast, Galar et al. [12] found significant differences in this respect (EUR 2927.3 in CG and EUR 1674.3 in IG; *p* ≤ 0.01). Patel et al. [3] defined ‘other costs’ as the sum of various associated costs, including clinical medicine, surgery, medical procedure unit, neurosurgery, oncology, organ transplantation, other ancillary, psychiatry, recovery room, and rehabilitation services. They reported lower costs in the IG (EUR 1307.5 in the CG and EUR 429.1 in the IG; they did not report statistical significance). Our costs are more consistent with those described by Galar et al. [12], probably because we defined ‘other costs’ similarly.

The discrepancy between the predicted cost reduction and the observed economic impact suggests the presence of a Type II error in the study design. While the sample size calculation targeted a reduction of EUR 3000 per patient, the actual decrease in economic costs was not statistically significant. This outcome indicates that the sample size, though calculated for 80% power and 95% confidence to detect the hypothesized effect, might not have captured smaller or variable cost reductions across groups. Furthermore, the large standard deviation in baseline costs highlights the potential influence of extreme values, which may have skewed the results and reduced the study’s power. A possible strategy to address this limitation in future analyses could involve truncating extreme values, such as the top and bottom 1%, through winsorization. This adjustment would help stabilize variance and better reflect central trends, reducing the impact of outliers on statistical calculations. While this method has limitations, such as the potential loss of information about high-cost cases, it offers a pragmatic approach to complementing a larger sample size or stratified analysis. Together, these refinements could minimize variability, reduce the risk of Type II errors, and ensure more robust and reliable evaluations of economic outcomes.

### Limitations

This study has several limitations that warrant discussion. First, the quasi-experimental design with a historical control group may have introduced biases stemming from unmeasured confounders or external changes over time, such as advancements in clinical practices or policy shifts, which could have influenced the outcomes. Second, while the sample size was calculated to provide sufficient statistical power (80%) to detect a significant EUR 3000 cost reduction, the high variability in economic data and the presence of extreme values likely contributed to the observed lack of significance. This suggests a potential Type II error that should be considered when interpreting the findings.

Additionally, the cost analysis primarily focused on direct hospital-related expenses, such as laboratory tests, imaging, and pharmacy, while omitting broader societal costs. These include patient out-of-pocket expenses, lost productivity, and caregiver burden, which are essential for a more comprehensive understanding of the economic implications of rapid microbiological diagnostics. Without incorporating these indirect costs, the actual economic impact of the intervention might be underestimated. Furthermore, the analysis did not account for potential long-term savings associated with earlier infection control and reduced antibiotic resistance due to optimized prescribing practices. These savings, while challenging to quantify in a short-term study, are crucial for evaluating the broader healthcare and societal benefits of such interventions.

In addition, the study was conducted in a single-center setting, which, while allowing for controlled data collection and analysis, restricts its applicability to other healthcare environments. Variations in healthcare resource availability, clinical workflows, and patient demographics across institutions may lead to different economic and clinical outcomes. For example, settings with higher baseline rates of diagnostic inefficiency or antimicrobial misuse might experience greater benefits from rapid diagnostic technologies. Conversely, highly optimized institutions might observe only marginal gains.

Thus, expanding future analyses to include multi-center studies across diverse healthcare settings would provide greater generalizability and help validate the findings under varying conditions. By addressing these limitations and incorporating a broader scope of economic impacts, future research can provide a more robust evaluation of the clinical and economic value of interventions like MALDI-TOF MS.

Most economic impact studies focus on total costs without a breakdown into different items or on the cost over the total time of admission, without considering specific data obtained from IPC, which makes direct comparison difficult. Dixon et al. [59], in their study of the impact of rapid identification by MALDI-TOF spectrometry, highlight the need for additional studies under real-world conditions, as the observed differences in cost and effectiveness between groups did not present conclusive data and may not accurately reflect the impact of MALDI-TOF MS technology in real clinical practice. Further studies are needed to establish a causal relationship between the effect of rapid microbiological information and its economic impact.

## 4. Materials and Methods

### 4.1. Setting

This study was conducted at the Clínica Universidad de Navarra (Pamplona, Spain), a 300-bed university medical center. The Clinical Microbiology Service conducted the investigation with the Infectious Diseases Service. An Infectious Diseases Clinician evaluated all patients.

The study population and intervention have been described previously [1]. We conducted a quasi-experimental pretest-posttest study with a historical control group (CG) and a prospective intervention group (IG) comprising patients with documented bacterial infections. The intervention involved implementing the MALDI-TOF MS system for bacterial identification, while the control group received conventional identification and antimicrobial susceptibility reporting. The current study focuses on analyzing the economic impact of this intervention by assessing the costs associated with additional diagnostic tests and antibiotic consumption.

All eligible patients were enrolled consecutively, with approximately one-third (31.7%) of the samples in both groups yielding positive urine cultures.

**Figure 1:**
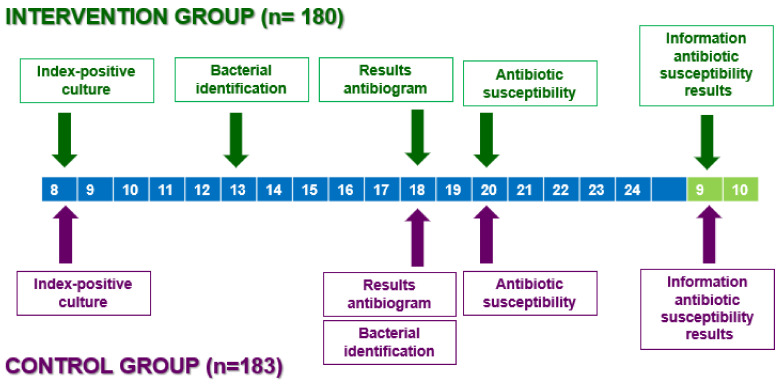
Algorithm for microbiological data, along with performance times observed in both the control and intervention groups. Green and purple arrows indicate activities for intervention and control groups, respectively, with key events annotated by day.

Three hundred sixty-three hospitalized patients with documented bacterial infections and confirmed bacterial isolates were included in a control group (CG) and an intervention group (IG). The CG consisted of 183 patients collected from June 2014 to December 2015. In this group, microbiological information on bacterial identification and antibiotic susceptibility was communicated to clinicians between 18:00 and 22:00 on the same day bacterial growth was detected (IPC). The IG included 180 patients collected from January 2016 to September 2017. In the IG, microbiological bacterial identification information was communicated to clinicians when it became available between 12:00 and 14:00 (rapid reporting), while antibiotic susceptibility data were provided between 18:00 and 22:00 (Figure 1).

This study utilizes a quasi-experimental design, precisely a single-group format with pre-test and post-test measures. It is both longitudinal and prospective. The CG corresponds to the pre-test phase (before intervention), while the IPC represents the post-test phase (after intervention).

In the CG, data were obtained via the Vitek^®^ 2 system (bioMerieux), enabling the identification of IPC and the antibiogram independently using the same device. In the IG, however, identification was conducted with the Vitek^®^ MS system (bioMerieux), which expedited the identification process. Antibiotic susceptibility data for both groups were recorded between 18:00 h and 22:00 h using Vitek^®^ 2 (bioMerieux). Samples were inoculated on specified media, such as blood agar, MacConkey, chocolate, and CPS for aerobic conditions and blood agar for anaerobic conditions in CG and IG.

For the CG, colonies grown on agar plates were transferred to sterile saline until a McFarland standard of 0.5 was achieved using an inoculation loop. This dilution facilitated identification with the Vitek^®^ 2 system (bioMerieux), utilizing identification cards for Gram-positive (GP) and Gram-negative (GN) bacteria. For antimicrobial susceptibility, cards AST-243 and AST-244 were used for Enterobacteriaceae, AST-245 for Pseudomonas and non-fermenting Gram-negative bacilli, AST-589 for Enterococcus, and AST-626 for Staphylococcus. Each card was sealed and placed in the Vitek^®^ 2 reader-incubator module (bioMerieux) at 35.5 °C, with results automatically analyzed and extracted from the database.

In the IG, identification was carried out using the Vitek^®^ MS system (bioMerieux), which analyses the protein spectrum generated by the bacteria (IPC) and compares it with existing protein profiles in the database, thus reducing identification times. Colonies grown on these media were deposited on the sample spots of the Flexi-Mass-DS TO-430 slide model (bioMérieux). One μL of Vitek^®^ MS-CHCA matrix (bioMérieux) was applied to each sample spot using a micropipette, and the mixture was air-dried until the matrix and sample crystallized. *E. coli* strain ATCC 8739 was used as the kit calibrator and applied to the central sample spots on the slide. The prepared slide containing all samples was then placed into the Vitek^®^ MS system to capture the mass spectra of each bacterial cell’s predominantly ribosomal proteins. The resulting data were analyzed using SARAMIS-KB- software (v4.17.0), which generated the spectra for each sample.

The Clinica Universidad de Navarra Ethics Committee approved the study under number 154/2014 on 8 January 2015.

### 4.2. Power Analysis and Sample Size Calculation

Sample size calculations were performed using Cytel Software’s Siz version 2.0 software based on data obtained by Galar et al. [12]. In their study, the mean cost per patient after early detection using current procedures was 12,402 euros, with a standard deviation of 11,087 euros. It was determined that a total of 338 patients, with 169 in each group, would be required to detect a reduction in mean expenditure per patient of 3000 euros (24%), with a confidence level of 95% and a statistical power of 80%. This power analysis confirmed that the study had sufficient power to identify statistically significant differences between groups, thereby reducing the risk of type II errors.

### 4.3. Statistical Analysis

The data analysis was conducted using IBM SPSS Statistics version 20. Descriptive statistics were computed for each variable, providing details for continuous variables such as mean, standard deviation (SD), maximum, minimum, and interquartile range (spanning from the first quartile, Q1, to the third quartile, Q3). For categorical variables, proportions were provided to give an overall understanding of the distribution of each category.

To evaluate data normality, three tests were applied: skewness, kurtosis, and the Shapiro–Wilk test. Skewness and kurtosis values were used to examine distribution symmetry and tail weight. A non-significant result from the Shapiro–Wilk test (*p* > 0.05) indicated normal distribution. Assumptions for each test were reviewed before analysis. Student *t*-tests were conducted for normally distributed data to compare the group mean. Non-parametric Mann–Whitney U tests were used for data that did not follow a normal distribution. These tests were selected as they fit the nature of our data and ensured more reliable results.

Chi-square tests were performed on categorical variables to identify significant associations between them. Statistical significance for all tests was established at *p* < 0.05, with all *p*-values being two-tailed. This approach reflects the non-directional nature of the tests, allowing for statistical significance to be detected in either direction of difference.

### 4.4. Variables Recorded Included

Demographic and clinical variables of the patients evaluated included age, sex, and severity of underlying disease according to McCabe–Jackson criteria [37] and CHI [38]. Other variables considered were severity of infection, service responsible, site of infection acquisition, positive index culture, and infectious syndrome.

A cost analysis was performed to determine the economic impact of the rapid information provided by the Clinical Microbiology Service compared to conventional information. The economic costs associated with both alternatives for patients (CG and IG) were calculated, considering direct fixed, direct variable, indirect, and total costs. Direct costs were calculated by estimating the resources required for each test, consultation, or intervention. Other CUN costs not included in these aspects (water, electricity, utilities, etc.) were considered indirect costs.

In the case of pharmacy products, a variable direct cost (the purchase price) was considered together with a fixed direct cost, which corresponds to the proportionate share of pharmacy staff. The total cost was disaggregated into different categories for analysis purposes, which are mutually exclusive. The term “non-laboratory examinations” included all tests performed on the patient, excluding those performed in the laboratory (radiology, ultrasound, computed tomography, magnetic resonance imaging, anatomical pathology, nuclear medicine). The “non-microbiology laboratory test” managed actions related to tests performed by the Laboratory Services, excluding those involving the Microbiology laboratory. The “microbiology laboratory test” performed all tests at the Clinical Microbiology Service. The cost of the stay was associated exclusively with the patient’s stay at the CUN. Other costs included clinical care, blood bank, surgery, and anesthesia. Pharmacy products addressed the total pharmaceutical cost and consumption of certain drugs, such as antibiotics, antifungals, antivirals, and other anti-infectives, including the costs of treatment with antiparasitics, antimycobacterial, vaccines, antiseptics, disinfectants, immune sera, and immunoglobulins.

All mean price values in euros for each patient in both groups were updated to 2017 using the CPI. The study assessed the cost of patient admission from the time of IPC to more accurately analyze the impact of early microbiological identification.

Analyses of response times were also performed, considering the time from the positive culture index to the delivery of the telephone report. In addition, the study recorded the time of sample collection, arrival, and processing, as well as the timing of telephone and automated reporting of identification and antimicrobial susceptibility test results.

## 5. Conclusions

Early communication of microbiological identification to clinicians was associated with reduced reporting time, contributing to a decrease in the number of patients undergoing complementary testing. This included significant reductions in the total number of patients requiring tests in the Microbiology, Biochemistry, Hematology, and Radiology Departments, as well as reductions in C-reactive protein and Anatomical Pathology tests. Furthermore, while rapid microbiological reporting was associated with a non-significant decrease in the economic costs of tests performed by the Microbiology Service, antibiotic consumption, and other related expenses, these differences can be attributed to the relatively short interval between identification and antimicrobial susceptibility testing.

This study highlights the potential of integrating rapid diagnostic technologies like MALDI-TOF MS into clinical practice to streamline diagnostic workflows, enhance decision-making efficiency, and reduce unnecessary testing. These findings underscore the importance of continued investment in advanced microbiological diagnostics and their integration with antimicrobial stewardship programs to optimize clinical outcomes and healthcare resource utilization. Further research is warranted to explore the long-term economic and clinical impacts of such interventions, particularly in settings with varying levels of antimicrobial resistance, patient severity, and resource availability.

## Figures and Tables

**Table 1:** Distribution of infectious syndromes in the study population.

	Total n = 363	Control Group n = 183	Intervention Group n = 180	*p*-Value †
Urinary, n (%)	137 (37.7)	74 (40.4)	63 (35.0)	0.28
Respiratory, n (%)	100 (27.6)	52 (28.4)	48 (26.7)	0.71
LRTI without pneumonia	67 (18.5)	29 (15.8)	38 (21.1)	0.20
Pneumonia	31 (8.5)	23 (12.6)	8 (4.4)	<0.01
URTI	2 (0.6)	0 (0)	2 (1.1)	0.15
Skin and soft tissue, n (%)	65 (17.9)	25 (13.7)	40 (22.2)	0.03
Abdominal, n (%)	41 (11.3)	24 (13.1)	17 (9.4)	0.27
Intra-abdominal	31 (8.5)	18 (9.8)	13 (7.2)	0.37
Biliary	10 (2.7)	6 (3.3)	4 (2.2)	0.55
Osteoarticular, n (%)	9 (2.5)	2 (1.1)	7 (3.9)	0.09
Endovascular, n (%)	5 (1.4)	4 (2.2)	1 (0.6)	0.22
Catheter-related infection	3 (0.8)	2 (1.1)	1 (0.6)	0.57
Endocarditis	2 (0.5)	2 (1.1)	0 (0)	0.16
Central nervous system, n (%)	2 (0.5)	1 (0.5)	1 (0.6)	0.76
No focus, n (%)	4 (1.1)	1 (0.5)	3 (1.7)	0.31

LRTI: Lower respiratory tract infection. URTI: Upper respiratory tract infection † Chi-Square Test.

**Table 2:** Economic evaluation from the index-positive culture (IPC) means and SD in euros (€).

	Total n = 363	Control Group n = 183	Intervention Group n = 180	*p*-Value †
**Complementary tests, mean ± SD (EUR)**	1098.3 ± 1443.6	1049.7 ± 1423.6	1146.9 ± 1453.2	0.52
Microbiology laboratory test	240.6 ± 291.5	245.1 ± 315.8	236.0 ± 265.1	0.76
Non-microbiology laboratory tests ^a^	235.2 ± 401.8	210.1 ± 350.5	260.8 ± 447.5	0.23
Non-laboratory examinations ^b^	640.1 ± 1062.3	630.3 ± 1030.8	650.1 ± 1096.2	0.85
**Pharmacy, mean ± SD (EUR)**	3258.4 ± 2004.6	3508.4± 2334.8	3198.5± 2606.8	0.23
Antibiotics	665.6 ± 1167.3	698.1 ± 1394.8	633.0 ± 859.1	0.59
Antifungals	496.3 ± 1550.7	424.9 ± 1550.7	567.8 ± 1681.7	0.41
Antivirals	64.9 ± 119.3	75.3 ± 134.8	57.8 ± 109.2	0.63
Other anti-infectives	10.5 ± 27.7	5.6 ± 7.4	13.72 ± 36.9	0.24
**Hospital stay, mean ± SD (EUR)**	6046.8 ± 10,104.5	5204.2 ± 6689.6	6899.4 ± 13,520.4	0.13
**Other costs ^c^, mean ± SD (EUR)**	2426.7 ± 4147.3	2480.6 ± 4563.3	2371.9 ± 3688.7	0.80
**TOTAL, mean ± SD (EUR)**	13,170.3 ± 9260.6	12,488.0 ± 8410.6	13,852.7 ± 8405.1	0.12

^a^ Laboratories other than CMS ^b^ Non-laboratory studies radiology, ultrasound, computed tomography, magnetic resonance imaging) ^c^ Costs associated with clinical care, blood bank, surgery, and anesthesia † Mann–Whitney U test.

**Table 3:** Economic evaluation from index-positive culture (IPC) median and range (minimum and maximum).

	Total n = 363	Control Group n = 183	Intervention Group n = 180	*p*-Value †
**Complementary tests, median (range)**	620.9 (7.3–15,523.2)	659.4 (7.3–6349.5)	601 (14.9–15,523.2)	0.52
Microbiology laboratory test	118.2 (14.9–1802.8)	128.6 (17.4–1802.8)	104.7 (14.9–1621.2)	0.76
Non-microbiology laboratory tests ^a^	87.1 (0–2789.7)	92.4 (0–2504.7)	84.6 (0–2789.7)	0.23
Non-laboratory examinations ^b^	282.5 (0–4543.2)	319.9 (0–4143.7)	210 (0–4543.2)	0.85
**Pharmacy, mean median (range)**	1616.7 (108.9–18,109.7)	1900.4 (138.4–18,109.7)	1470.1 (108.9–17,541.2)	0.23
Antibiotics	300 (13.1–7400.7)	250.1 (19.8–7400.7)	313.8 (13.1–6652.2)	0.59
Antifungals	8.5 (0–11,367.2)	7.7 (0–11,367.2)	11.2 (0–7816.9)	0.41
Antivirals	17.8 (0–551)	26.1 (0–551)	19 (0–465.2)	0.63
Other anti-infectives	5.8 (0–88.8)	4.3 (0–32.5)	8.8 (0–88.8)	0.24
**Hospital stay, median (range) (EUR)**	2728.4 (172.4–76,253.3)	2664.2 (172.4–54,517.3)	2759 (172.4–76,253.3)	0.13
**Other costs ^c^, median (range) (EUR)**	1131.7 (42.4–35,495.6)	1414.4 (42.4–32,307.9)	967.6 (63.4–35,495.6)	0.80
**TOTAL, median (range) (EUR)**	979,293.2 (484.7–82,040.7)	9713.6 (484.7–62,994.1)	9087.9 (1071.3–82,040.7)	0.12

^a^ Laboratories other than CMS ^b^ Non-laboratory studies radiology, ultrasound, computed tomography, magnetic resonance imaging) ^c^ Costs associated with clinical care, blood bank, surgery, and anesthesia † Mann–Whitney U test.

## Data Availability

All data are available and stored in the informatics system of our hospital, Clínica Universidad de Navarra.

## Supplementary Materials

The following supporting information can be downloaded at: https://www.mdpi.com/article/10.3390/antibiotics13121163/s1, Table S1: Number and percentage of patients undergoing complementary tests from the IPC.
