# Supplementary material for: Accelerated Bacterial Identification with MALDI-TOF MS Leads to Fewer Diagnostic Tests and Cost Savings
Source: Antibiotics (Basel). 2024 Dec 2;13(12):1163. doi: 10.3390/antibiotics13121163 (PMC11672624; doi:10.3390/antibiotics13121163)
Supplement: Supplementary file 1 [file antibiotics-13-01163-s001.zip › antibiotics-3282763-supplementary.pdf]

Table S1. Number and percentage of patients undergoing complementary tests from the IPC.

	Total<br>n=363	Control Group<br>n=183	Intervention Group<br>n=180	p-value
**Microbiology laboratory test, no. (%)**	**229 (63.1)**	**136 (74.3)**	**93 (51.7)**	**0.01**
Blood culture	141 (38.8)	75 (41.0)	66 (36.7)	0.40
Urine culture	149 (41.0)	81 (44.3)	68 (37.8)	0.21
Cultures of wounds and abscesses	76 (20.9)	33 (18.0)	43 (23.9)	0.17
Culture of respiratory samples	101 (27.8)	53 (29.0)	48 (26.7)	0.62
Clostridioides difficile	50 (13.8)	26 (14.2)	24 (13.3)	0.81
Stool cultures	50 (13.8)	26 (14.2)	24 (13.3)	0.81
Culture of biological fluids	32 (8.8)	20 (10.9)	12 (6.7)	0.15
Biopsy cultures	17 (4.7)	9 (4.9)	8 (4.4)	0.83
Other microbiological cultures	20 (5.5)	11 (6.0)	9 (5.0)	0.68
**Non-microbiology laboratory test, no. (%)**	**306 (84.3)**	**161 (88.0)**	**145 (80.6)**	**0.05**
Biochemical laboratory tests, no. (%)	306 (84.3)	161 (88.0)	145 (80.6)	0.05
Ionogram	281 (77.4)	147 (80.3)	134 (74.4)	0.18
C Reactive Protein	275 (75.8)	148 (80.9)	127 (70.6)	0.02
Procalcitonin	106 (29.2)	56 (30.6)	50 (27.8)	0.55
Urea	245 (67.5)	126 (68.8)	119 (66.1)	0.57
Creatinine	264 (72.7)	136 (74.3)	128 (71.1)	0.49
Other biochemical tests	293 (80.7)	153 (83.6)	140 (77.8)	0.16
Hematological laboratory tests, no. (%)	330 (90.4)	172 (94.0)	158 (85.6)	0.04
Hemogram	298 (82.1)	157 (85.8)	141 (78.3)	0.06
Other hematology tests, [a]	123 (33.9)	67 (36.6)	56 (31.1)	0.27
**Non-laboratory examinations, no (%)**	**251 (69.1)**	**142 (77.6)**	**109 (60.6)**	**0.01**
Imaging examinations<br>X-ray, no. (%)	180 (49.6)	100 (54.6)	80 (44.4)	0.05
Chest X-ray	132 (36.4)	74 (40.4)	58 (32.2)	0.10
Abdominal X-ray	27 (7.4)	14 (7.6)	13 (7.2)	0.87
Other X-ray	99 (27.3)	55 (30.0)	44 (24.4)	0.23
Ultrasound, no. (%)	95 (26.2)	53 (29.0)	42 (23.3)	0.22
Abdominal ultrasound	45 (12.4)	20 (10.9)	25 (13.9)	0.39
Other ultrasound	66 (18.2)	37 (20.2)	29 (16.1)	0.31
CT [b], no. (%)	72 (19.8)	44 (24.0)	28 (15.5)	0.04
Chest CT	43 (11.8)	25 (13.7)	18 (10.0)	0.28
Abdominal CT	47 (12.9)	30 (16.4)	17 (9.4)	0.05
Other CT	15 (4.1)	9 (4.9)	6 (3.3)	0.46
MRI [c], no. (%)	28 (7.7)	14 (7.6)	14 (7.8)	0.96
Chest MRI	1 (0.3)	1 (0.5)	0 (0)	0.50
Abdominal MRI	1 (0.3)	0 (0)	1 (0.6)	0.49
Other MRI	26 (7.2)	13 (7.1)	13 (7.2)	0.96
Anatomical pathology tests, no. (%)	11 (3.0)	10 (5.5)	1 (0.6)	0.01
Nuclear Medicine, no. (%)	5 (1.4)	2 (1.1)	3 (1.7)	0.67
Cardiovascular examinations, no. (%)	97 (26.7)	56 (30.6)	41 (22.8)	0.09
Respiratory examinations, no. (%)	20 (5.5)	9 (4.9)	11 (6.1)	0.62
Gastroenterologist examinations, no. (%)	47 (12.9)	20 (10.9)	27 (15.0)	0.25
Medical consultation, no. (%)	165 (45.4)	86 (47.0)	79 (43.9)	0.27

[a] Include reticulocyte count, clotting time, hematocrit, and serum protein electrophoresis [b] Computed tomography [c] Magnetic Resonance Imaging
